# Adaptive clinical trials: key concepts for clinical researchers

**DOI:** 10.36416/1806-3756/e20260412

**Published:** 2026-09-14

**Authors:** Victor Zuniga Dourado, Cecilia Patino, Juliana Carvalho Ferreira

**Affiliations:** 1. Methods in Epidemiologic, Clinical, and Operations Research-MECOR-program, American Thoracic Society/Asociación Latinoamericana del Tórax, Montevideo, Uruguay.; 2. Departamento de Ciências do Movemento Humano, Laboratório de Epidemiologia, Movimento Humano e Saúde Digital - EPIMOV-e - Universidade Federal de São Paulo - UNIFESP - Santos (SP) Brasil.; 3. Lown Scholars Program, Department of Global Health and Population, Harvard T.H. Chan School of Public Health, Boston (MA) USA.; 4. Department of Preventive Medicine, Keck School of Medicine, University of Southern California, Los Angeles (CA) USA.; 5. Divisão de Pneumologia, Instituto do Coração, Hospital das Clínicas Faculdade de Medicina, Universidade de São Paulo, São Paulo (SP) Brasil.

## PRACTICAL SCENARIO

Researchers created a smartphone app to help adults stay physically active, using goal setting, self-monitoring, and personalized messages. In a pilot study, some participants quickly increased their daily step counts in response to motivational messages, whereas others remained inactive despite using the app regularly.

Instead of giving everyone the same intervention throughout the study, it might make more sense to adapt the intervention according to each participant’s response. For example, participants who do not initially improve their physical activity level could receive additional support, such as gamification, group activities, or other strategies, whereas those who respond well could be continue with the original approach or receive a less intensive intervention. However, clinical trials typically do not allow for this type of flexible strategy.^1^ ^,^ ^2^

## WHY CONSIDER ADAPTIVE CLINICAL TRIALS?

Traditional randomized clinical trials are the gold standard for assessing the impact of interventions on patient outcomes. In these studies, the research plan is set before the study begins and remains unchanged throughout the trial. This approach works well for many research questions, but it can be limiting when participants respond very differently to the same intervention or when investigators realize that the intervention should be modified, but the study is already ongoing. 

Adaptive clinical trials were developed to address these challenges. Unlike traditional clinical trials, they allow planned changes during the study based on interim results while still maintaining scientific and statistical standards. These planned changes might include adjusting the sample size, changing how participants are assigned to groups, stopping early for benefit or harm, or adding and removing treatment groups in platform trials.^3^

For example, during the COVID-19 pandemic, adaptive designs enabled the rapid addition of promising treatments and the discontinuation of those that were ineffective, which helped accelerate scientific discovery.

## FROM THE ADAPTATION OF THE STUDY TO THE ADAPTATION OF THE INTERVENTION

Let’s go back to our practical scenario. If some participants become more active after receiving self-monitoring of their daily step count, activity goals, and personalized messages, but others do not, using the same intervention for everyone may not answer the research question. In clinical practice, researchers and health professionals want to know when to continue with a strategy that is working and when to change or strengthen the intensity of the intervention if results are not satisfactory.

The researchers used this response-based approach in that study. Participants received different combinations of behavior-change techniques, such as step tracking, goal setting, personalized messages, and gamification. They measured participants’ progress by changes in step counts over time. Those who responded well continued with the original strategy, whereas those who did not improve their daily step counts were assigned to alternative strategies, such as additional gamification or social support.^1^ Figure 1 illustrates the difference between maintaining the same intervention for all study participants and adapting the intervention according to each participant’s response during the study.

**Figure 1:**
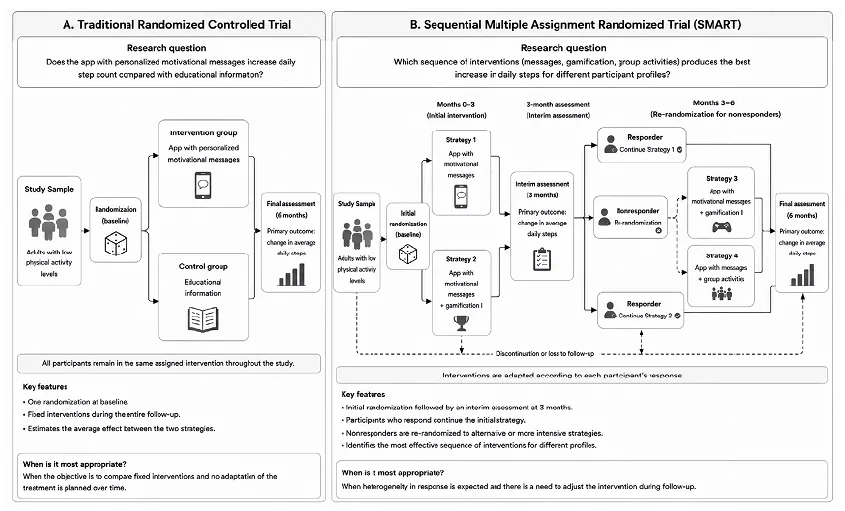
Comparison between a traditional randomized controlled trial and a Sequential Multiple Assignment Randomized Trial (SMART) for developing physical activity interventions.

Adapted from Simoes et al.^1^ and Morais Pereira Simões.^2^

A. Traditional randomized controlled trial: Participants are randomized at baseline to a fixed intervention or control condition and remain in the assigned group throughout follow-up. This design estimates the average effect of one predefined intervention compared with another condition but does not allow the intervention to be adapted according to participants’ responses during the study.

B. SMART: participants are initially randomized to different intervention strategies and undergo a prespecified intermediate assessment of their response. Responders continue their initial strategy, whereas nonresponders are re-randomized to alternative or intensified strategies. This design allows investigators to determine which sequence of interventions is the most effective according to participants’ responses and can therefore inform the development of adaptive interventions. Dashed arrows indicate possible discontinuation or loss to follow-up.

Instead of just asking “Does the intervention work?”, researchers can also ask, “Is it working for this participant?” and, if not, “What should we try next?” The response observed during follow-up is not just a result; it can also help guide the next steps outlined in the study plan.

This approach is especially helpful when people are expected to respond differently to treatment. In the practical scenario study, about half of the participants did not respond initially, whereas those who did respond continued to increase their step counts over time. These findings highlight that a single fixed strategy may not work for everyone and that monitoring participants’ progress during the study may help identify those who need a different or more intensive approach. In practice, this design can support stepped interventions: start with simple strategies and add more intensive ones only when needed (Figure 1). 

Adaptive clinical trials do not replace traditional randomized trials, but they provide additional design options when participants are expected to respond differently or when adaptations may be needed during follow-up. By using prespecified rules for making planned changes, these designs can make clinical trials more closely reflect real-life decision-making: start an intervention, assess how it works, and then decide whether to continue, change, or intensify it.

## KEY POINTS

- Adaptive clinical trials allow researchers to make prespecified changes to the trial during its implementation while maintaining scientific and statistical standards.
- These designs are especially useful when participants are expected to respond differently to treatment or when prespecified changes to the intervention need to be made during the study.
- Researchers can use participants’ responses during the study to decide whether an intervention should remain unchanged, be modified, or be intensified.
- Thus, beyond asking “Does the intervention work?”, an adaptive approach can also help answer “For whom does it work, and what should we do if it does not?’

## References

[1] Simoes MDSMP, Proença NL, Lauria VT, do Nascimento MB, Padovani RDC, Dourado VZ (2026). Effects of Using a Smartphone App Combined With Behavior Change Techniques on the Level of Physical Activity Among Adults and Older Adults Sequential Multiple Assignment Randomized Trial. J Med Internet Res.

[2] Morais Pereira Simões MDS, de Barros Gonze B, Leite Proença N, Tonon Lauria V, Demarchi Silva Terra V, da Costa Padovani R, Zuniga Dourado V (2019). Use of a smartphone app combined with gamification to increase the level of physical activity of adults and older adults protocol of a sequential multiple assignment randomized trial. Trials.

[3] Ryan EG, Couturier DL, Heritier S (2022). Bayesian adaptive clinical trial designs for respiratory medicine. Respirology.

